# Developing authentic assessment instrument for fundamental forehand and backhand groundstroke techniques using an actions-based method

**DOI:** 10.1016/j.heliyon.2024.e26203

**Published:** 2024-02-19

**Authors:** Ngatman, M. Furqon Hidayatullah, Sugiyanto, Sapta Kunta Purnama

**Affiliations:** a Physical Education, Sport, Health, and Recreation Study Program, Faculty of Sport Science, Yogyakarta State University, Indonesia; b Sport Science Study Program, Faculty of Sport, Sebelas Maret University, Indonesia

**Keywords:** Authentic assessment, Forehand and backhand groundstroke, Actions method

## Abstract

This research aims to produce an authentic assessment for fundamental forehand and backhand groundstroke techniques, using the actions method of learning outcomes in tennis courses, for students at Yogyakarta State University's (FIK UNY) Faculty of Sports and Health Sciences. This study employed development research with a 4D model (Define, Design, Development, and Disseminate). This research was implemented through the preliminary research stage, designing assessment instruments, and validating the instrument model developed. In this study, 30 students were chosen to sample using a purposive sampling strategy, with 5 experts serving as validators. Data was collected through questionnaires and expert validation with the Delphi technique. The collected data is analyzed and concluded. Based on the analysis of the collected data, it can be concluded that authentic assessment instruments of fundamental forehand and backhand groundstrokes techniques using the Actions Method are needed to evaluate the learning objectives of tennis courses for students at FIK UNY. This assessment model can be used to evaluate the learning outcomes of tennis courses for FIK UNY students. It makes it easy for lecturers to evaluate students' learning outcomes authentically, has excellent validity, and has a high rater reliability coefficient.

## 1 Introduction

One of the most important aspects of learning is assessment that cannot be dissociated from it in higher education. To determine which model of assessment tool is best for evaluating student learning outcomes, there needs to be an instrument that can access all learning outcomes performance in a real/authentic manner for the cognitive, affective, and psychomotor realms. Authentic assessments are those that can be used to evaluate every student's learning outcome [ [1]]. An authentic assessment involves giving students a task that pushes them to show how they interact with the real world as a significant reflection of the knowledge and abilities they have gained. To add, authentic assessment is a model of assessment that can measure real and significant learning outcomes of students in the domains of attitudes, knowledge, and skills [ [[2], [3], [4]]]. By using this kind of assessment model, we will get a comprehensive and factual picture of students' abilities. Thus, a comprehensive or all-encompassing approach to evaluating student learning outcomes that refers to the "real" world is known as authentic assessment.

At Yogyakarta State University's (FIK UNY) Faculty of Sports and Health Sciences, tennis is taught as each of the courses offered in every study program. Course lecturers always conduct an assessment at end of each practical tennis lecture to measure how far to which students have met the learning objectives. One of the fundamental techniques that is assessed is the forehand and backhand groundstroke technique since these two techniques belong to essential fundamental tennis techniques that must be comprehended. The two most common groundstroke techniques in tennis are the forehand and backhand [ [[5], [6], [7], [8]]].

Tennis represents one of the sports with complicated motion characteristics since the game of tennis falls under the category of open motor abilities (open skills). As a result, for each stroke (forehand and backhand groundstroke), we must examine four steps in the open skill process of the actions method: perception, decision, execution, and feedback. These four phases include the actions method's primary features in today's modern tennis teaching [ [9]]. In performing forehand and backhand groundstroke, the four stages of the action method are the inseparable stages to achieve success in every stroke [ [[10], [11], [12], [13], [14]]].

A framework for using "what to accomplish/what to do by tennis players" is the main focus of the actions approach, which, for a tennis player, is always tightly tied to "how to do" The actions method is a tennis teaching approach that offers a structure to combine and strengthen each open skill phase [ [15]]. The actions approach is a way of teaching tennis for beginners which employs the open skill process to build perception, decision, execution, and feedback skills [ [8,16,17]]. Likewise, beginning players in tennis can learn fundamental techniques employing the actions approach, which adapts instruction to the needs of the open skill stage of the match [ [18]].

Presently, lecturers of tennis courses at Yogyakarta State University (FIK UNY) face some challenges. These include (a) assessments conducted by lecturers regarding the outcomes of learning fundamental forehand and backhand groundstroke techniques, and (b) students continuing to use the sports skill test, which was created by foreign experts several decades ago. The created sports skill exam did not gauge student performance concerning actual events in tennis playing conditions; rather, it was restricted to examining the degree of proficiency in fundamental skills. The sports skill test utilized to implement the assessment model is a predictor deemed "invalid" in assessing students' playing abilities [[19]]. Furthermore, FIK UNY currently has yet to have a common evaluation tool that may be applied to evaluate how effectively students are being taught the fundamentals of forehand and backhand groundstroke.

Investigating the issues raised earlier, the researcher proceeded to perform a needs analysis using questionnaire tools and student interviews with tennis course graduates from all four study programs. A standard assessment tool that can be used to evaluate the actual performance of student forehand and backhand groundstroke learning outcomes is needed by the Study Programs (Sports Coaching Education, Sports Science, Health Physical Education and Recreation, Primary Teacher Education, and Physical Education) at FIK UNY, according to the results of the search conducted through questionnaires and interviews.

The author conducted research to develop an authentic assessment instrument for tennis learning outcomes for FIK UNY students, taking into account the background of the problem and the significance of assessment instruments to access the learning outcomes of basic technical skills playing tennis for students authentically and holistically.

This research aims to create an authentic tool model for assessing fundamental forehand and backhand groundstroke skills based on the action-based learning outcomes of FIK UNY students' tennis courses. These major objectives are subsequently subdivided into three distinct goals, including.

➢ Perform preliminary research on the requirement for authentic assessment tools for fundamental forehand and backhand groundstrokes based on the actions method learning results of FIK UNY tennis courses.
➢ Develop a draft tool using the actions method to accurately evaluate fundamental forehand and backhand groundstroke techniques, and
➢ Conduct a validation analysis of the draft tools using the actions approach that has been created to authentically assess fundamental techniques of forehand and backhand groundstrokes.

## 2 Research methods

### 2.1 Research design and sample

The research design used in this study was development studies employing a 4D model (Define, Design, Development, and Disseminate), simplified into three stages of activity, namely: 1) conducting preliminary research, 2) drafting assessment instruments, and 3) validating drafts of assessment instruments that have been designed.

The number of research participants used to develop the authentic assessment model was 30 students using a purposive sampling technique with details comprised of 12 female and 18 male students. There are five experts total—three male experts and two female experts—who are involved in becoming validators. The number of students involved as the research sample was only 30 students because the requirement was that students had already taken tennis courses (purposive sampling).

Participants in the development of an authentic assessment model have the following characteristics.

− 30 students who have taken tennis courses (purposive sample).
- The research participants' age range was 19–22 years.
- Had at least one year of tennis experience.
- Had a basic understanding of tennis.
- Be able to act as a sample/research subject.

### Ethical approval

This study received approval from the Institute of Research and Community Service at Universitas Negeri Yogyakarta's Ethics Committee, Indonesia (B/26.5/UN34.21/TU/2021) on February 9, 2021. All participants signed written informed consent forms. The questionnaires were anonymized, and patients were permitted to decline participation in the study at any time whenever they were uncomfortable.

### 2.2 Data collection and study instrument

The research was conducted using activities, including preliminary studies through observation of the assessment model so far given by lecturers who teach courses and the study of literature to obtain a theoretical foundation. The preliminary research that the researchers did include making observations during the assessment process carried out by the lecturers teaching the previous course, where the assessment model carried out by the lecturers was only oriented towards mastering basic techniques and did not describe the real playing situation. The assessment carried out was subjective because it did not use standard/standardized instruments. Furthermore, the researcher gave a questionnaire to the lecturer teaching the field tennis course to explore information about the assessment of student tennis skills that had been done so far. In addition, researchers also conducted interviews with students who had taken field tennis courses related to the tennis skills assessment model used by students. On the basis of this preliminary research, it was utilized as a basis for developing an authentic assessment model based on the action method.

To add, the type of questionnaire implemented in this study was closed (used for preliminary studies and needs analysis), and the types of answers included yes and no options (for five tennis lecturers as the respondents) with a category scale of 1–5 (assessment criteria 5 = Very Relevant, 4 = Relevant, 3 = Doubtful, 2 = Not Relevant, and 1 = Very Not Relevant). Furthermore, when developing the authentic assessment model, the researchers used the assessment rubric which was applied by expert assessors to observe fundamental technical abilities for the forehand and backhand groundstroke using the tennis actions method when playing with ½ competition using a 1–9 rating scale and a 1–3 rating scale (assessment criterion 3 = essential, 2 = useful but not essential, and 1 = not useful) for content validity assessment.

To increase the reliability and validity of this instrument, the indicators of the assessment rubric instrument were retrieved as follows: Self-Confidence and Mental Play, Before Hitting (Stage I & II Actions Method: Perception and Decision), When Execution of Punches and Evaluation of Blow Results (Stage III & IV Actions Method: Execution and Feedback), Attitude and Behavior While Playing.

There are several important points in the instrument that we made, namely.

a. to gather information on whether the tennis lecturers evaluate students' knowledge of the fundamentals of both forehand and backhand groundstroke techniques at the end of each tennis class,
b. to seek the assessment instruments used standard or not,
c. to assess whether the assessment aspects used are carried out whether it includes mastery of techniques, application of tactics, and students' attitudes in playing tennis (student performance-based assessments),
d. to evaluate the assessment instruments used by lecturers whether they still use sport skill tests made by foreign experts or created/modified by themselves, and whether assessment instruments used are based on techniques and tactics according to the characteristics of the actual game of tennis.

The tool used to assess the fundamental groundstroke technique for the forehand and backhand based on the actions’ method and the assessment of learning outcomes for the basic forehand and backhand groundstroke technique carried out by the lecturers were examined for their objectivity, and whether it required a standard assessment instrument based on the actual situation of playing tennis so that tennis players can develop perception, decision, execution, and feedback abilities.

The development stage of the assessment instrument started with researchers compiling authentic assessment instruments that are oriented towards the fundamental techniques of forehand and backhand groundstroke in the game of tennis based on actions method. At the development stage of this performance-based appraisal instrument, an assessment rubric focused on motion components was created using fundamental techniques that reflected the characteristics of the actual tennis playing situation. Thus, at this stage, a set of performance-based instruments for learning tennis had been obtained.

After developing an authentic assessment tool for the fundamental groundstroke techniques of the forehand and backhand, the following steps taken by the researcher were to conduct a validation test by a material expert in tennis and continue with the validation by experts who were competent in the field of preparing tests, measuring and evaluating Sports Physical Education and Health (PJOK). Based on input from the validation of the two experts (expert judgment), revisions and improvements were made to the authentic assessment. The expert assessment stage employed the Delphi technique where the experts provided an assessment and advice on the assessment instrument model developed, by filling out a questionnaire given by the researcher.

### 2.3 Statistical analysis

The professionals who evaluated the drafting assessment instrument included five academics, including a field tennis expert, a Test, Measurement, and Evaluation expert, and a health assessment evaluation expert. Testing for content validity involved evaluating expert judgment through subjective ratings, and the results were examined using the CVR (Content Validity Ratio) formula [19]:

CVR = $\left[\;2\;\frac{Mp}{M}\right]$- 1

Information:

Mp = ∑Expertswhogivegoodmarks(3−4votes).

M = Number of experts.

1 = Results that experts do.

Reliability testing of authentic assessment instrument model development was performed using Consistency Alpha Cronbach's chassis [ [20]]. Cronbach's Alpha Consistency test provides a requirement that a model can be said to be reliable if the calculation results show a value of >0.75. In the next stage, we determined the reliability among raters employing the Interclass Correlation Coefficient (ICC) [ [20]].

## 3 Research results

### 3.1 Conduct preliminary study

The results of the preliminary study of the assessment system that has been used in FIK can be looked in Table 1.

**Table 1:** Results of preliminary analysis.

No.	Components	Percentage	
		Yes	No
1	Do you conclude each tennis course lecture by evaluating the fundamental technical abilities of the forehand and backhand groundstrokes?	100	0
2	Does your performance-based assessment of fundamental forehand and backhand groundstroke techniques consider student attitudes towards playing tennis as well as technique mastery and tactic implementation?	40	60
3	Have you ever used standardized testing tools to evaluate the fundamentals of the forehand and backhand groundstroke?	40	60
4	Do you use the fundamental groundstroke assessment instruments for the forehand and backhand as tools for self-improvement and modification?	20	80
5	Do you utilize instruments designed by international or local professionals to examine fundamental techniques for groundstrokes, both forehand and backhand?	80	20
6	Do you use foreign or local experts who are focused on the technique's mastery level to create the fundamental technique of forehand and backhand groundstrokes for your sports skill tests?	60	40
7	Do you utilize process and product assessments to evaluate your basic forehand and backhand groundstroke techniques?	20	80
8	Do you employ criteria of achievement for the process and products that students demonstrate to evaluate the fundamentals of the forehand and backhand groundstroke?	20	80
9	Do you need a basic technique assessment instrument for forehand and backhand groundstroke oriented to technical ability and tactics based on actions method?	100	0
10	Do you need a standard assessment instrument for authentic assessment of fundamental forehand techniques and backhand groundstroke-based actions methods to assess tennis learning outcomes for students?	100	0

This assessment is essential for assessing student learning outcomes and determining their level of basic forehand and backhand groundstroke engineering skills. Thus, lecturers can ensure that students have the necessary skills to play tennis effectively and efficiently. These assessments also assist in learning planning for the next meeting, so that lecturers can ensure that each student receives learning that suits his or her needs and skill level. Overall, these assessments perform a crucial part in the process of learning and ensure that students acquire the necessary skills in tennis courses. By conducting regular assessments, lecturers can ensure that students get effective and quality learning.

From the information provided, it is evident that the evaluation of fundamental groundstroke technical abilities, both forehand and backhand, is an essential component of the tennis course lectures at the Department of Sports Science, Yogyakarta State University. This is confirmed by the results of the research obtained, where 100% of respondents said that lecturers who teach courses always conduct an assessment at the end of each lecture. The conclusion of this information is that lecturers who teach tennis courses in most cases do not assess the basic techniques of forehand and backhand groundstroke of students by covering aspects of mastery of techniques, implementation of tactics, and student attitudes in playing tennis (performance-based assessment). The results showed that 60% of lecturers stated that they did not conduct performance-based assessments, while 40% of lecturers had carried out these assessments.

Additionally, 60% of the respondents said they did not employ conventional assessment tools to evaluate students' mastery of fundamental forehand and backhand groundstroke skills, while 40% of assessments were carried out using standard assessment instruments. 80% of tennis courses stated that the fundamental assessment tools for the forehand and backhand groundstroke techniques used by lecturers are product assessment instruments of foreign or local experts, while 20% are instruments resulting from their development/modification. From the results of questionnaires and interviews with lecturers teaching Field Tennis, 60% of lecturers stated that they used forehand and backhand groundstroke basic technique assessment instruments that referred to foreign expert tennis sport skill test instruments oriented to the level of mastery of techniques, while 40% of other lecturers did not use foreign expert tennis sport skill test instruments.

From the opinion of the lecturers of tennis courses, 20% stated that forehand and backhand groundstroke basic technique assessment instruments rely on the evaluation of products and processes while 80% of the criteria were based on the product (final result) only. Of the results of questionnaires and interviews, 100% stated that playing tennis requires basic technique assessment instruments for forehand and backhand groundstroke tennis oriented to technical and tactical abilities based on actions methods. From the lecturers who teach tennis courses, 100% stated that it requires a common evaluation tool that accurately evaluates fundamental forehand and backhand groundstroke skills using the actions method.

The results showed that the majority of lecturers who teach tennis courses do not assess the outcome of mastering fundamental groundstroke skills for both the forehand and backhand, implementation of tactics, and student attitudes in playing tennis. The majority of assessment instruments used are product assessment instruments from foreign experts and do not use assessment instruments that are developed/modified by lecturers themselves. The product (final result) is the sole basis for most assessment tools, and it also serves as the basis for evaluating the fundamental groundstroke skills of the forehand and backhand. According to all respondents, standard tools for assessment were required for fundamental forehand and backhand groundstrokes based on action methods focused on technical skills and strategies.

### 3.2 Drafting the assessment instrument

A preliminary draft of the authentic assessment instrument was created based on the findings of the needs analysis through preliminary investigations, literature reviews, and relevant research results. The proposed authentic assessment tool comprises two components: (1) an open skill process grid that lists fundamental forehand and backhand groundstroke techniques based on actions method; (2) an observation sheet that includes guidelines for evaluating these fundamental techniques, and (3) a scoring sheet for final performance assessment of student tennis learning outcomes. A draft grid of instruments for authentic assessment of fundamental forehand techniques and backhand groundstrokes based on actions methods compiled to obtain expert assessment is presented in Table 2.

**Table 2:** Instrument grids for authentic assessment of fundamental techniques for both forehand and backhand groundstrokes based on action method.

Basic Techniques	Component	Indicator
Forehand and Backhand Based Groundstroke Actions Method	Self-Confidence and Mental Play (Psychological Aspects)	●Have an abundance of confidence when doing forehand and backhand groundstrokes ●Make decisions with bravery and without hesitancy ●Have mentally obstinate when it comes to important things. ●The playing plan is implemented at that precise moment. ●Have incredibly motivated to win the game.
	Before Hitting (Stage I & II Actions Method: Perception and Decision)	●The capacity to assess game conditions; ●Adapt to the ball's arrival ●Assess position before hitting the ball. ●Precision in making decisions prior to forehand strikes and backhand groundstroke volleys. ● 5 Ball control skills: predict the direction, speed, spin, altitude, and ball's distance or depth. ● Attitude-taking skills: ready position, balance, backswing, point of contact, and persistence
	When Execution of Punches and Evaluation of Blow Results (Stage III & IV Actions Method: Execution and Feedback)	● When striking the groundstroke with the forehand and backhand, decisions are made accurately, successfully, and efficiently. ● Making good decisions during forehand and backhand groundstrokes makes it harder for the opposition to return the ball, and these decisions can help score points. ●being aware of the advantages and disadvantages of every deliberate groundstroke punch, including forehand and backhand.
	Attitude and Behavior While Playing	● After hitting the forehand and backhand groundstroke always return to the starting position (basic position) before hitting. ● During the game, they still show a high consistency of spirit. ● Makes signals of disappointment after striking groundstrokes with the forehand and backhand when an unexpected fault happens. ● Demonstrates that the character always plays with fair play and sportsmanship. respects the opponent while they play

### 3.3 Validating the design of the drafted assessment instrument

For the next stage of the research, validation tests are carried out by Five experts have been designated to provide guidance and input using the Delphi Technique. Expert validation is carried out so that the designed authentic assessment model is ready for field testing. The results of the assessment and the advice of experts are essential to refine the draft so that it can be piloted in the field. The experts who assessed the draft assessment instrument consisted of five people with a background as an academic, including a field tennis expert, a Test, Measurement and Evaluation expert, and a health assessment evaluation expert.

The observation sheet and authentic assessment guidelines consist of four factors that are used as observation guidelines to assess self-confidence and mental play/aspects of psychology, so far In addition to their attitudes and behaviors when playing, students can apply the fundamental stages of forehand and backhand groundstroke based on actions method (perception ability, decision, execution, and feedback). As research subjects, students are assigned to run tennis matches using a 1/2 competitive system. A pro-set tiebreak is the match system that is employed, with a score of 10 indicating victory. The assessment was carried out using observation sheets and authentic assessment guidelines.

In the next stage after the developed authentic assessment instrument model has a level of accuracy of assessment, the researcher tests the validity of the content of the developed assessment model. In offering an assessment of the components of the researcher's assessment tool, Table 3 displays the three potential answers that were given to the assessors: vital/important, useful but not crucial, and useless. The assessment data of the five assessors can be read in Table 3.

**Table 3:** The outcome of the tennis expert's value.

Assessment	Factor Assessment			
	Self- Confidence and Mental Play (1)	Before Hitting (Stage I & II Actions Method: Perception and Decision) (2)	When Execution of Punches and Evaluation of Blow Results (Stage III & IV Actions Method: Execution and Feedback) (3)	Attitude and Behavior While Playing (4)
**AAL**	Crucial	Crucial	Crucial	Crucial
**HYL**	Crucial	Crucial	Crucial	Crucial
**YDT**	Crucial	Crucial	Crucial	Crucial
**WSN**	Crucial	Crucial	Crucial	Useful but not crucial
**AGS**	Useful but not crucial	Crucial	Crucial	Crucial

Note: AAL, HYL, YDT, WSN, AGS is acronym for 3 male experts and 2 female experts.

Based on the expert assessment's results being summarized in Table 5, for factor 1 (Self-Confidence and Mental play), four appraisers state it is crucial and 1 appraiser states it is useful but not crucial. For factor 2 (Before Hitting), five expert appraisers state it is essential. For factor 3 (When Execution of Punches and Evaluation of Blow Results), five assessors state it is crucial. For factor 4 (Attitudes and Behavior while playing tennis), four expert appraisers state it is crucial, whereas one appraiser states it is useful but not crucial. From this data, Table 4 presents the results of an analysis of this data using the Content Validity Ratio (CVR) formula.

**Table 4:** CVR calculated the content validity results.

Instrument Factors	Content Validity Coefficient
Self-Confidence and Mental Play (Psychological Aspects)	0.60
Before Hitting (Stage I & II Actions Method: Perception and Decision)	1.00
When Execution of Punches and Evaluation of Blow Results (Stage III & IV Actions Method: Execution and Feedback)	1.00
Attitude and Behavior While Playing	0.60

**Table 5:** Coefficient reliability test of the four components of an authentic assessment tool.

Factor	*Consistency Alpha Cronbach*	*ICC (Anava*-*General Multifacet Model)*	Condition
Self-Confidence and Mental Play (Psychological Aspects)	0.862	0.874	Reliable
Before Hitting (Stage I & II Actions Method: Perception and Decision)	0.892	0.910	Reliable
When Execution of Punches and Evaluation of Blow Results (Stage III & IV Actions Method: Execution and Feedback)	0.883	0.864	Reliable
Attitude and Behavior While Playing	0.861	0.893	Reliable

All factors in the developed assessment instrument have a content validity coefficient › 0.50. Thus, based on the actions method, these characteristics can be utilized as reliable assessment tools to evaluate fundamental forehand and backhand groundstroke techniques.

Because there were more than two raters in this study, ICC analysis was utilized for the reliability testing among raters. Researchers employed this ICC technique to determine the degree of stability or consistency among raters when assessing students' tennis playing abilities. Table 5 comprises the findings of the reliability test conducted on the four components of the authentic assessment tool.

Thus, if utilized as an instrument model for authentic assessment of basic technique, these four criteria have a high degree of determination (reliability).

## 4 Discussion

In order to enhance the data collection process, assessment instruments' validity and reliability are necessary. Any method of collecting data must be valid and reliable in order to be effective [ [21]]. The instruments employed must have strong validity and reliability to raise the quality of data collection. Absolute validity and reliability are essential conditions for the measurement and evaluation of the psychomotor domain. Similarly, the validity and reliability requirements of assessment instruments must be taken into account while developing assessments, particularly performance-based assessments (authentic assessments).

Four components of the assessment rubric which can be applied as authentic assessment instruments for fundamental forehand techniques and backhand groundstroke-based actions method have been successfully developed by several field tennis experts who specialize in developing authentic assessment instruments. These factors were derived from the results of data analysis through content validation tests. The forehand and backhand groundstroke methods are regarded as fundamental skills because they are very significant basic techniques that must be mastered when playing tennis. Success in hitting forehand and backhand groundstrokes is largely determined by the tennis player's capability to examine the direction, trajectory, and preparing for the ball to arrive, to decide before making a stroke/decision, to make strokes based on the decisions made/execution, and to receive feedback on each stroke made [ [[10], [11], [12], [13],^15^,^17^]].

As an assessment tool, the content validity test findings for the four criteria have a fairly good validity coefficient, indicating some degree of accuracy. The coefficient of Content Validity Ratio (CVR) for each of the factors and indicators is shown to be approximately >0.50. For students enrolled in tennis courses at FIK UNY, the elements and indicators of these instruments can serve as authentic assessment tools for learning objectives related to the fundamental forehand and backhand groundstrokes.

This is in line with the findings [ [22]], who said that an assessment tool has a good level of validity if the content validity coefficient (CVR) reached >0.50. To put it another way, the instrument can measure the items that need to be measured and has a certain level of measurement accuracy [ [23]]. had a similar view, stating that an assessment tool has accurately captured the intended construct to be tested if it has a high coefficient of content validity. Moreover [ [24]], contend that an instrument can be deemed valid in terms of content if all of the construct or variable to be measured is included in its elements. According to this opinion, FIK UNY students' learning outcomes for tennis courses can be assessed authentically using the four components of the proposed actions approach and the fundamental forehand technique.

After analysis, authentic assessment tools for fundamental forehand and backhand groundstroke techniques based on actions method obtained a high-reliability coefficient through both Inter-Tester Reliability (Anava-General Multifacet Model r = 0.864–0.910) and Genova Programme analysis (Consistency Alpha Cronbach r = 0.861–0.892). It implies that: (1) the developed authentic assessment tool has a degree of consistency/determination of assessment among the raters in evaluating the same subject; (2) all five raters agree that these four factors are crucial and essential for the usefulness of fundamental groundstroke techniques, both forehand and backhand; and (3) Based on the actions method of tennis course learning outcomes for FIK UNY students, authentic assessment tools are accepted and can be utilized as instruments to measure fundamental forehand and backhand groundstrokes. According to Strand, an instrument is suitable for use as an evaluation tool [ [25]] to assess a sport's skills, including tennis, if it has an interrater and an interrater reliability coefficient of ≥0.80 [ [26]]. asserted that a test fulfills the criteria for being a competent assessment test or instrument if it measures "to what extent the tool used for measuring evaluates consistently the measured targets."

Winning a game of tennis requires more than just being proficient with the forehand and backhand groundstrokes. Having mastered both fundamental techniques is not the only factor that matters [ [11]]. lists the following as contributing factors to the success of a player's forehand and backhand groundstroke in tennis: competitive mindset, the capacity to assess situations and evaluate the direction in which the ball is moving (perception), decision making (decision), the accuracy of the hit (execution), providing feedback on the punch's outcome (feedback), and performing attitudes and behaviors. Additionally, playing tennis requires a quick movement response because all of these aspects happen swiftly and concurrently [ [11]]. The Canada Coaches Association had a similar viewpoint, stating that players of tennis must constantly react and make decisions in a variety of circumstances. The process of reacting begins with scenario analysis, followed by predicting and modifying the direction in which the ball is going to arrive in decision-making, technical decision-making, and the assessment of the blow's effects. As a result, it is anticipated that the training given will enable players to automate decision-making and modify it under technical judgments.Based on professional judgment, these elements have to be included in the assessment while creating the instrument.

The learning outcomes of the ½ competition match system tennis skills will be closely controlled to determine how self-confidence and mental play in the game, anticipation of decision-making, decision-making accuracy during punch execution, and a tennis player's attitude and behavior while playing are applied in the four aspects of fundamental groundstroke and forehand techniques. Players will experience the real tennis playing atmosphere during this first half of the competition, which they will always confront when they play [ [27]]. says that given the modification in the circumstances, playing tennis during a match will increase a student's cognitive engagement, spark their interest, enable them to play more games, and provide them with the chance to apply concepts from one game to another. An assessment sheet and an observation sheet are required in order to analyze the shown performance and to be able to closely monitor all four factors.

Thus, the assessment instrument model will be appropriate and effective if we consider the shortcomings and benefits of the assessment system that has been used, and lecturers require an effective model to appraise the overall results of learning basic forehand and backhand groundstroke technical skills. According to the above description, this study has research limitations, specifically that it should include more research samples (>30 samples) to broaden the generalization of the research (and the evaluation instruments produced are not only confined to students).

## 5 Conclusion

Assessing the learning objectives of tennis courses for FIK UNY students entails the use of authentic assessment instruments for fundamental forehand strokes and the backhand groundstroke based on the actions method. It has been found that lecturers may more easily assess student learning outcomes of tennis courses when they employ the instrument model for authentic assessment of fundamental forehand technique and backhand groundstroke based on actions approach. To evaluate the learning objectives of tennis courses for FIK UNY students, an authentic assessment tool through good validity and a high rater reliability coefficient might be employed.

It is hoped that this research will assist readers or other researchers who seek to evaluate students' ability levels in line with actual tennis playing situations by employing the authentic assessment tool of fundamental forehand and backhand groundstroke techniques based on this action method. Due to the development of this model of an authentic assessment instrument, it can be applied as a guideline and prototype for developing authentic assessment instruments for other basic techniques and advanced playing techniques.

The fact that this study was limited to FIK UNY students and did not include any other populations raises the possibility that the findings would differ for other populations. Therefore, more research is needed to test this assessment model in other populations. Therefore, the opportunity for future research is to be able to test authentic assessment models of fundamental techniques both forehand and backhand groundstroke based on action methods in other populations such as other university students or even school students. In addition, it can also test this scoring model in other sports courses such as badminton or football to expand the application of this scoring model in the field of sports.

## Ethical statement

Thereby, Ngatman, M. Furqon Hidayatullah, Sugiyanto, and Sapta Kunta Purnama, the authors, consciously guarantee that for the manuscript titled “Developing Authentic Assessment Instrument for Fundamental Forehand and Backhand Groundstroke Techniques Using an Actions-Based Method” the following is accomplished. 1) The writing presented here is the writers' own original creation, never before published. 2) No other publications are currently considering to publish the paper.

## Data availability statement

Data will be made available on request.

## Additional information

No additional information is available for this paper.

## CRediT authorship contribution statement

**Ngatman:** Writing – original draft, Project administration, Methodology, Investigation, Formal analysis, Conceptualization, Writing – original draft, Project administration, Methodology, Investigation, Formal analysis, Conceptualization. **M. Furqon Hidayatullah:** Writing – review & editing, Validation, Methodology, Formal analysis. **Sugiyanto:** Writing – review & editing, Supervision, Methodology, Formal analysis, Writing – review & editing, Supervision, Methodology, Formal analysis. **Sapta Kunta Purnama:** Writing – review & editing, Supervision, Methodology, Formal analysis.

## Declaration of competing interest

The authors declare that they have no known competing financial interests or personal relationships that could have appeared to influence the work reported in this paper.
